# Cisplatin Uptake in Macrophage Subtypes at the Single-Cell Level by LA-ICP-TOFMS Imaging

**DOI:** 10.1021/acs.analchem.1c03442

**Published:** 2021-11-30

**Authors:** Anna Schoeberl, Michael Gutmann, Sarah Theiner, Martin Schaier, Andreas Schweikert, Walter Berger, Gunda Koellensperger

**Affiliations:** † Institute of Analytical Chemistry, Faculty of Chemistry, University of Vienna, Waehringer Strasse 38, 1090 Vienna, Austria; ‡ Institute of Cancer Research and Comprehensive Cancer Center, Medical University of Vienna, Borschkegasse 8A, 1090 Vienna, Austria; § Institute of Inorganic Chemistry, Faculty of Chemistry, University of Vienna, Waehringer Strasse 42, 1090 Vienna, Austria

## Abstract

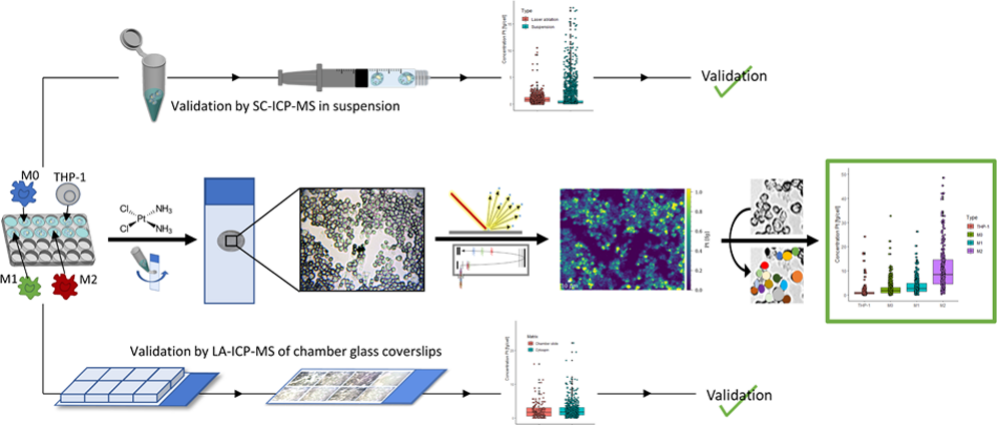

A high-throughput laser ablation–inductively coupled plasma–time-of-flight mass spectrometry (LA-ICP-TOFMS) workflow was implemented for quantitative single-cell analysis following cytospin preparation of cells. For the first time, in vitro studies on cisplatin exposure addressed human monocytes and monocyte-derived macrophages (undifferentiated THP-1 monocytic cells, differentiated M0 macrophages, as well as further polarized M1 and M2 phenotypes) at the single-cell level. The models are of particular interest as macrophages comprise the biggest part of immune cells present in the tumor microenvironment and play an important role in modulating tumor growth and progression. The introduced bioimaging workflow proved to be universally applicable to adherent and suspension cell cultures and fit-for-purpose for the quantitative analysis of several hundreds of cells within minutes. Both, cross-validation of the method with single-cell analysis in suspension for THP-1 cells and with LA-ICP-TOFMS analysis of adherent M0 cells grown on chambered glass coverslips, revealed agreeing platinum concentrations at the single-cell level. A high incorporation of cisplatin was observed in M2 macrophages compared to the M0 and M1 macrophage subtypes and the monocyte model, THP-1. The combination with bright-field images and monitoring of highly abundant endogenous elements such as phosphorus and sodium at a high spatial resolution allowed assessing cell size and important morphological cell parameters and thus straightforward control over several cell conditions. This way, apoptotic cells and cell debris as well as doublets or cell clusters could be easily excluded prior to data evaluation without additional staining.

## Introduction

Single-cell analysis based on inductively coupled plasma–mass spectrometry (ICP-MS) allows investigating heterogeneous cell populations with regard to cell numbers, cell types, and functionality.^1,2^ Over the last decade, elemental single-cell analysis of cells in suspension^3−9^ has evolved to the established strategy of mass cytometry, successfully applied in clinical routines. More recently, the potential of imaging strategies by laser ablation (LA)-ICPMS was recognized and has triggered a wave of exciting studies.^10−16^ Newest technologies of low-dispersion laser ablation setups have enabled the analysis of single cells and (sub-)cellular imaging (with spot sizes down to 1 μm) at pixel acquisition rates of >200 Hz.^17,18^ Time-of-flight mass spectrometers (TOF-MS) offer ideal platforms to assess multiple isotopes/elements within short transient signals, generated either by the introduction of single cells in suspension (300–400 μs) or by low-dispersion laser ablation setups (low ms regime).^17,19^ To date, single-cell analysis of cells in suspension remains unrivaled in terms of throughput. It is the state-of-the-art to perform multiplex analysis of up to 1000 cells/s in mass cytometry.^20,21^ Barcoding strategies allow us to analyze multiple samples within one analytical run.^22−25^ As a drawback, information on cell morphology and cell size, resulting in different transport efficiencies is precluded and needs to be addressed for unbiased analysis of a heterogeneous cell population. The state-of-the-art of single-cell analysis enabled by ICP-MS (including mass cytometry) is summarized elsewhere.^1,2^ Compared to single-cell analysis in suspension, bioimaging methods reveal the spatial arrangement of cell populations, which is an essential information. In fact, the cross-talk of proximal cells is accepted as a major factor dynamically driving cell functionality and cell states. In addition to proximity, cell size and cell morphology can be easily assessed via combination with microscopic images or with nondisruptive techniques (prior to LA).^26,27^ As a major drawback, today, most LA-ICPMS studies cover only the investigation of comparably low cell numbers, hampering statistical evaluation and thus biological interpretation. Therefore, method-oriented studies are of paramount importance, focusing on advancement of spatial resolution,^10,18^ throughput,^17,19^ and quantification strategies,^28−30^ as well as on the evaluation of endogenous elemental patterns as cell markers.^11,31,32^

Concerning sample types, LA-ICPMS imaging of single cells has covered tissue samples,^33^ cell smears (or cell thin films),^11^ chambered glass coverslips with cells directly growing on them,^13^ cell arrays produced by a piezo-acoustic microarrayer,^34^ and cytospins,^14−16^ where cells are deposited on glass slides through centrifugal forces. In tissue sections, the heterogeneous and very dense cell population poses a challenge for the detection of single-cell boundaries. Cell smears represent a well-suitable matrix, but it has to be paid attention to the shear forces produced by the preparation of cell smears, which can easily damage cells. Analysis of chambered glass coverslips represents the approach where minimal sample preparation is required, but which is limited to adherent cell models. Moreover, only tight control of confluence ensures monolayers of separated single cells. The production of cytospins allows the concentration of cells on a small given area with an even distribution, the possibility of producing multiple slides per sample,^35^ and the simple removal of the culture media during production.^12,35^ Evidently, the analysis of tissue samples at single-cell resolution requires more advanced imaging/staining strategies to resolve single cells. Managh et al. implemented cytospins of human CD4+ T cells addressing the uptake of Gd-based contrast agents for magnetic resonance imaging (MRI) by LA-ICPMS. Cytospins were used for cell deposition, and full ablation of the cells was achieved using a spot size larger than the diameter of the cells. The uptake was assessed in a qualitative manner considering several hundreds of cells.^12^ The method was applied for in vivo tracking of rare cells, and in a subsequent study, also Au-tagged human regulatory macrophages were investigated in immunodeficient mice.^36^ Using blood smear samples of patients undergoing cisplatin chemotherapy, qualitative multielement analysis of several hundred single blood cells enabled differentiation of cell types based on LA-ICP-TOFMS and unsupervised statistical analysis.^11^ One study reported on single-cell arraying of THP-1 cells and potential quantitative LA-ICP-TOFMS analysis using microdroplet standards.^34^ Recently, multimodal imaging studies have emerged for single-cell analysis.^37^ In this context, alveolar macrophages exposed to nanoparticles were investigated by synchrotron radiation micro X-ray fluorescence spectroscopy (SR-μXRF) and LA-ICPMS. This multimodal study showed that quantitative nanoparticle accumulation in vitro was comparable to in vivo samples.^26^ SR-XRF and LA-ICPMS were also combined to study the quantitative Cu uptake in around 100 single cells of a model organism.^28^ Van Acker et al. showed the potential of hybrid labels carrying fluorophores and metal labels enabling the combined use of confocal fluorescence microscopy and LA-ICPMS on the same sample. The expression levels of two receptor proteins were quantitatively assessed by LA-ICPMS in breast cancer cells grown on glass chamber slides.^13^ Imaging mass cytometry (IMC) combines high-resolution laser ablation and ICP-TOFMS detection to perform highly multiplexed single-cell analysis on tissue sections that are stained with metal-conjugated antibodies at (sub-)cellular resolution. A pioneering study by Giesen et al. showed the possibilities of this new method by mapping 32 proteins^38^ in tumor sections after labeling with lanthanide tags using a 1 μm beam. Subsequently, the potential of IMC was shown for a vast number of applications in the clinical context.^39,40^

This work introduces a validated workflow capable of the quantitative multielement analysis in several hundreds of single cells at unprecedented throughput (few minutes) using a low-dispersion LA setup in combination with ICP-TOFMS detection. This fundamental study will focus on monocytes and monocyte-derived macrophage subtypes exposed to the clinically used chemotherapeutic drug cisplatin. Monocyte-derived macrophages are mononuclear phagocytes of the myeloid origin and represent an important part of the innate immune response as first-line defense against pathogens. They are characterized by high phagocytic and secretory activity, distinct plasticity, and a marked dynamic responsiveness to changes in the microenvironment. An imbalance of distinct macrophage subtypes is implicated in cancer, and tumor-associated macrophages (TAMs) represent the most abundant tumor-infiltrating immune cell type.^41−43^ Knowledge regarding the effect and response of macrophages and macrophage subtypes toward metal-based anticancer drug chemotherapy is limited, and dissection at the single-cell level is required to elucidate the heterogeneity of polarized macrophage subtypes regarding drug uptake, accumulation, and intracellular distribution.^44−46^

## Experimental Section

### Chemicals and Reagents

Ultrapure water (18.2 MΩ cm, ELGA Water purification system, Purelab Ultra MK2, U.K.) was used for all dilutions for ICP-MS measurements. A multielement stock solution and single-element standard solutions were purchased from Labkings (Hilversum, The Netherlands). The transport efficiency of the single-cell ICP-MS introduction system was determined using the EQ Four Element Calibration Beads (Fluidigm, San Francisco, CA) and 100 nm colloidal gold nanoparticles (mean diameter 101.2 nm, 5.60 × 10^9^ particles mL^–1^) (BBI Solutions, U.K.). Gelatin (from cold-water fish skin) was obtained from Sigma-Aldrich (Vienna, Austria). A Cell-ID Intercalator-Ir (125 μM), a Cell-ID Intercalator-Rh (500 μM), an anti-pH2AX ^165^Ho-labeled antibody, and a Maxpar Fix and Perm Buffer were purchased from Fluidigm (San Francisco, CA). The target retrieval solution (pH 9) for antigen retrieval was purchased from Agilent Technologies (Waldbronn, Germany).

Sample preparation (except cell culture) and all ICP-MS measurements were carried out in clean room ISO class 5 and 4, respectively. All cell culture media and reagents were purchased from Sigma-Aldrich (St. Louis, MO), and all plastic dishes, plates, and flasks were obtained from StarLab (Hamburg, Germany) unless stated otherwise. Cisplatin was synthesized at the Institute of Inorganic Chemistry, University of Vienna, according to literature procedures.^47^ TrypLE Express with phenol red (Gibco, Fisher Scientific, Roskilde, Denmark) was used for gentle cell detachment from culture plastic following the instructions of the manufacturer.

### Cell Culture

See the Supporting Information for details concerning cell culture experiments.

### SC-ICP-TOFMS

Single-cell ICP-MS analysis of THP-1 cells (in suspension) was performed on an *icp*TOF 2R ICP-TOFMS instrument from TOFWERK AG (Thun, Switzerland). The cell samples were introduced using a single-cell introduction system from Glass Expansion (Port Melbourne, Australia) consisting of a concentric glass micro-nebulizer and a low volume, on-axis spray chamber, where a sheath gas is installed to achieve less cell deposition and higher transport efficiencies. A syringe pump (Spetec GmbH, Erding, Germany) with a low-volume syringe (Hamilton Company, Reno, NV) was used to provide a constant flow of 10 μL min^–1^. The measurements were performed in time-resolved analysis using an integration time of 3 ms. The ICP-TOFMS was optimized daily to achieve highest intensities while keeping the oxide level and doubly charged ratio below 5%. The standard operation mode was used, which balances mass resolving power, sensitivity, and ion transmission across the entire mass range and which allows the analysis of ions from *m*/*z* = 14 to 256. The instrument was equipped with a torch injector with an inner diameter of 2.5 mm and nickel sampler and skimmer cones with a skimmer cone insert of 2.8 mm in diameter. A radio-frequency power of 1550 W, an auxiliary Ar gas flow rate of 0.80 L min^–1^, and a plasma Ar gas flow rate of 14 L min^–1^ were used. The nebulizer gas flow was set to 0.40 mL min^–1^ and the additional Ar gas flow rate was set to 45% of the maximum flow rate provided by the internal mass flow controller of the instrument. Instrumental parameters are summarized in Table S1.

### LA-ICP-TOFMS

All laser ablation measurements were carried out with an Iridia 193 nm laser ablation system (Teledyne Photon Machines, Bozeman, MT) coupled to an *icp*TOF 2R ICP-TOFMS instrument (TOFWERK AG, Thun, Switzerland). The laser ablation system is equipped with an ultrafast ablation cell^18^ in the cobalt ablation chamber and the aerosol rapid introduction system (ARIS). The ARIS was used to introduce an Ar makeup gas flow (∼ 0.90 L min^–1^) into the optimized He carrier gas flow (0.60 L min^–1^) before entering the plasma. The laser ablation settings and ICP-TOFMS settings were optimized on a daily basis to achieve high intensities for ^26^Mg^+^, ^59^Co^+^, ^115^In^+^, and ^238^U^+^ while keeping the oxide level (based on ^238^U^16^O^+^/^238^U^+^) below 2% and the laser-induced elemental fractionation (based on ^238^U^+^/^232^Th^+^) around 1. A NIST SRM612 glass-certified reference material (National Institute for Standards and Technology, Gaithersburg, MD) was used for optimization. Laser ablation was performed using a square spot size of 5 μm, a fixed dosage of 2, and the line scans were overlapping in the y-direction by 2.5 μm, which resulted in a pixel size of 2.5 × 2.5 μm^2^. A repetition rate of 200 Hz and a fluence of 0.60 J cm^–2^ were used for complete ablation of the biological material and the gelatin micro-droplet standards without ablating the glass. The standard operation mode was used, which balances mass resolving power, sensitivity, and ion transmission across the entire measured mass range and which allows the analysis of ions from *m*/*z* = 14 to 256. The integration and read-out rate were optimized to match the laser ablation repetition rate. Instrumental parameters for LA-ICP-TOFMS measurements are summarized in Table S1.

### Data Acquisition and Processing of ICP-TOFMS Data

Data were recorded using TofPilot 1.3.4.0 (TOFWERK AG, Thun, Switzerland) and saved in the open-hierarchical data format (HDF5, www.hdfgroup.org). Post-processing of the data was performed in Tofware v3.2.0, a TOFWERK data analysis package, which is used as an add-on on IgorPro (Wavemetric, Inc., OR). The data processing included the following steps: (1) drift correction of the mass peak position in the spectra over time via time-dependent mass calibration, (2) determining the peak shape, and (3) fitting and subtracting the mass spectral baseline. The data were exported as CSV files.

### Data Processing of the Single-Cell Analysis in Suspension

SC-ICP-TOFMS data were further processed using an in-house-written R-script. For the purpose of extracting cell events from the background signal, a previously published iterative procedure was used,^49,50^ but adapted for a Poisson distributed signal, since for low count rates, the noise is no longer normal distributed, instead it can more closely be described as Poisson noise.^51,52^ The following threshold was used
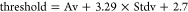
1where Av is the average concentration of all events of one sample and Stdv is the standard deviation of the same events. First, all events containing ^195^Pt^+^ signals above this threshold were marked as single-cell events and removed from the dataset. With the remaining data points, a new threshold was set again and the procedure was repeated until no new cell events above the threshold were found. The approximate transport efficiency of the cells was calculated using metal-labeled polystyrene beads (Fluidigm). Quantification of Pt in the cell events was performed by external calibration using liquid platinum standards according to eq 2 ^3^
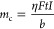
2where *m*_c_ is the mass of the element in the cell, η is the transport efficiency of the liquid standards, *F* is the sample flow, *t* is the integration time, *I* is the intensity of the isotope (after subtracting the background), and *b* is the slope of the calibration curve. For the liquid standards, a transport efficiency of 100% was assumed, which was already proven for such low flow rates in a study by Stefánka et al.^53^

### Data Processing of LA-ICP-TOFMS Analysis

The LA-ICP-TOFMS data were further processed in HDIP-v1.5.5, a laser ablation software provided by Teledyne Photon Machines (Bozeman, MT). First, the bright-field images of the area of interest prior to ablation were aligned with the signal intensity maps of ^31^P obtained by LA-ICP-TOFMS analysis. The bright-field images were used for cell segmentation, where microscope images are split into segments containing individual cells. Finally, the selected areas were transferred to the aligned signal intensity maps and the sum intensity and the equivalent diameter were exported for each isotope and segment.

Quantification of LA-ICP-TOFMS data was based on a multipoint calibration using gelatin-based microdroplet standards as described by Schweikert et al.^29^ Briefly, a CellenONE X1 micro-spotter and cell arrayer (Cellenion, Lyon, France) was used to produce arrays of gelatin microdroplets of around 400 ± 5 pL (resulting in droplet diameters of around 200 μm), containing multielement standard solutions onto glass slides. After spotting, the droplets dry within seconds due to their small size. The gelatin droplets were quantitatively ablated by LA-ICP-TOFMS, and the sum of the elemental signal intensities was extracted via HDIP and used for external calibration.

## Results and Discussion

### Validation of the Cytospin-LA-ICP-TOFMS Method

**Figure 1:**
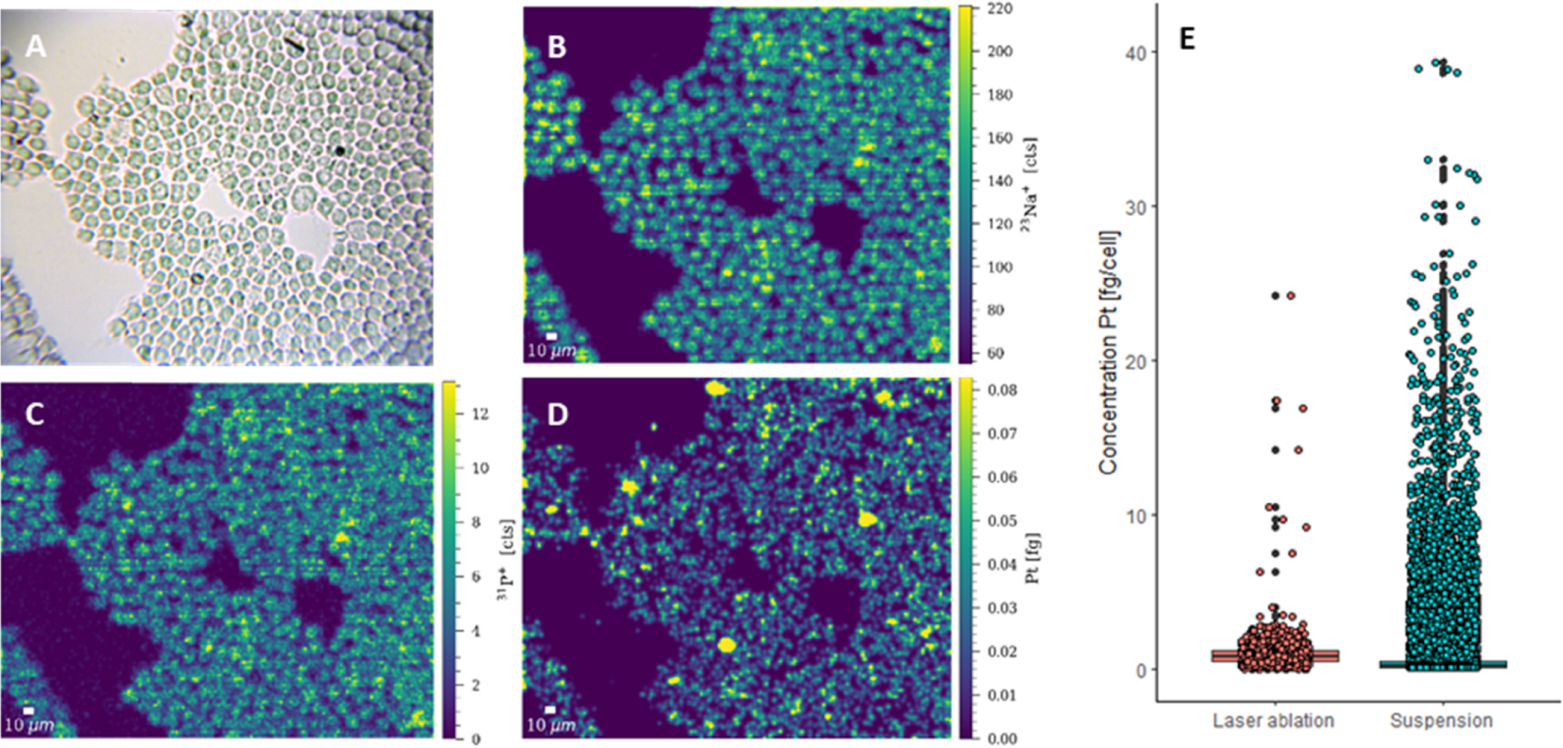
(A) Bright-field image of THP-1 cells prepared by cytospin prior to ablation. Signal intensity maps of (B) ^23^Na^+^ and (C) ^31^P^+^, obtained by LA-ICP-TOFMS imaging. (D) Map of the total amount of Pt (fg) in THP-1 cells after treatment with 10 μM cisplatin for 6 h. The following laser ablation parameters were used: square laser spot size of 5 μm, fixed dosage mode of 2, repetition rate of 200 Hz, and the parallel lines overlapped one another by 2.5 μm. (E) Box plot depicting the concentration of Pt in THP-1 cells treated with 10 μM cisplatin for 6 h obtained by LA-ICP-TOFMS (orange) or SC-ICP-TOFMS analysis (blue). The validation is based on ∼1000 cells for LA-ICP-TOFMS and ∼20 000 cells for solution-based analysis.

Studying cellular uptake and accumulation of metal-based anticancer drugs at the single-cell level in a quantitative manner demands for thorough validation as trueness bias arising either from cellular manipulations prior to analysis and/or from cell size effects in the introduction system can potentially jeopardize the accuracy. Therefore, in this study, cross-validation experiments were performed comparing quantitative single-cell analysis as obtained by orthogonal LA-ICP-TOFMS imaging of single cells deposited on surfaces and single-cell analysis in suspension. The investigated methods relied on independent calibration strategies. For LA-ICP-TOFMS analysis, quantification was enabled by the use of gelatin-based microdroplet standards, as described previously.^29^ The human monocytic THP-1 cell line upon cisplatin exposure was selected as it represents a widely used in vitro model to study macrophage differentiation and polarization toward different activation states. In comparison to primary human monocyte-derived macrophages isolated from peripheral blood, THP-1 cells exhibit no variation between different donors and are therefore ideally suited to reproducibly investigate the characteristics of macrophage subtypes.^44^ Being a suspension culture, the sample preparation steps for solution measurements could be minimized. In the case of imaging, cytospin deposition was addressed. This method enables the deposition of a monolayer of well-confined cells from a cell suspension onto a defined, circular area on a slide resulting in an even distribution of the cells enabling straightforward automated data evaluation. Cytospins are widely applied in cytology, histology, and hematology, but only a few studies have discussed the use of cytospins for LA-ICPMS.^14−16^ Especially, to the best of the author’s knowledge, its application for in vitro studies on metal-based anticancer drugs is novel. Figure 1A represents a bright-field image of deposited THP-1 cells (exposed to cisplatin), visualizing their morphology and size. LA-ICP-TOFMS imaging of several hundreds of cells was performed within minutes at a spatial resolution of 2.5 × 2.5 μm^2^ (Figure 1B,C,D). The rapid measurements were followed by automated data evaluation. The implemented software together with the high spatial resolution enabled by the low-dispersion LA setup allowed automated recognition of cells and cellular boundaries based on bright-field images and subsequent alignment of microscopy images with LA-ICPMS-related signal intensity maps (using abundant elements, here ^31^P). After setting cell-specific parameters including lower and upper diameters, minimum circularity, and minimum convexity, the sphere equivalent diameter of each cell together with the intensity integral over the selected areas was calculated. For the investigated THP-1 cells, a diameter in the range of 15–20 μm was observed based on their sodium and phosphorus contents (Figure 1B,C). As a key advantage, the high throughput of the LA-ICP-TOFMS approach enabled to measure sample replicates and calibration routines comparable to liquid ICP-MS analysis based on multipoint gelatin microdroplet standards.^29^

For the investigated cell model, a limit of detection of 0.012 fg platinum per cell was calculated for LA-ICP-TOFMS analysis (using the method by Longerich et al.^54^). The calculation considered the LOD for individual pixels multiplied by the average number of pixels per cell. It has to be taken into account that in the scale of the LA-ICP-TOFMS images, the amount of Pt per pixel is shown and not the Pt amount per cell resulting in lower values. For suspension analysis, external calibration of platinum relied on liquid standards.^3^ Therefore, the Pt amount per cell was inferred from the signal height of the single-cell events calibrated by liquid standards and assuming a transport efficiency of 100%, which is a typical value for that low flow rate when using a full consumption introduction system. The transport efficiency of polystyrene beads with a diameter of 4 μm was assessed to report on the actual cell number bias. Typically, a share of 20% was successfully transported to the plasma. The LOD was calculated to 0.066 fg platinum per cell event, being in the same order of magnitude as the LOD assessed for LA-ICP-TOFMS analysis. One major disadvantage of single-cell analysis in suspension is that this method suffers from size-dependent transport efficiencies, leading to a discrimination of larger cells.^2,55^ While the cell size can be directly assessed in high-resolution bioimaging experiments, dedicated labeling strategies^56,57^ are employed to infer information on cell size for single-cell analysis in suspension. However, in this study, the narrow size distribution of the THP-1 cells allowed omitting this step and thus facilitated the cross-validation of the two orthogonal approaches. Only platinum detection served for cell event assignment. As can be seen in Figure 1E, both methods were in good agreement with regard to platinum levels assessed in the THP-1 cell model. The values ranged from 0.46 to 1.22 fg cell^–1^ and from 0.12 to 0.54 fg cell^–1^ (25–75 percentile) for LA-ICP-TOFMS analysis and solution-based ICP-TOFMS analysis, respectively. A Welch two-sample *t*-test was applied to test if the mean of group A (laser ablation ICP-TOFMS analysis) is equal to the mean of group B (suspension analysis). The test showed that the difference was not statistically significant (*p*-value = 0.0504). The validation was based on ∼1000 cells for laser ablation and ∼20 000 cells for solution-based analysis. As a result of the integration time of 3 ms in solution measurements, most likely double cell events could not be excluded,^5^ explaining the observed cell events with higher platinum levels and thus larger distribution as compared to bioimaging experiments.

**Figure 2:**
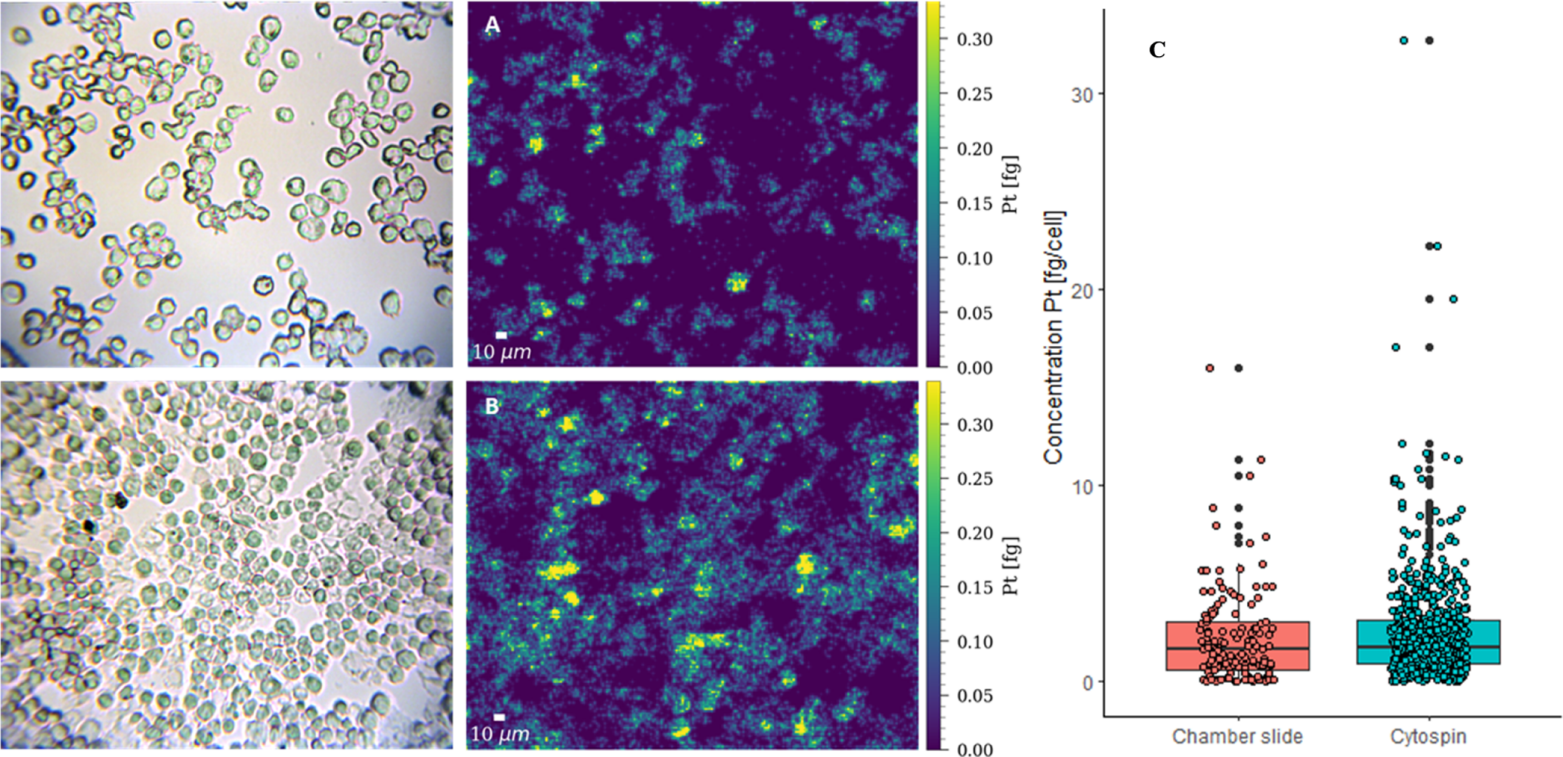
Bright-field images (left) and maps of the total amount of Pt (fg) obtained by LA-ICP-TOFMS imaging (right) of M0 macrophages, based on (A) chambered glass coverslips and (B) cytospins. The following laser ablation parameters were used: square laser spot size of 5 μm, fixed dosage mode of 2, repetition rate of 200 Hz, and the parallel lines overlapped one another by 2.5 μm. (C) Box plot showing the concentration of Pt in M0 macrophages treated with 10 μM cisplatin for 6 h obtained by LA-ICP-TOFMS analysis of chambered glass coverslips (left) and cytospins (right). The validation is based on ∼200 cells for chambered glass coverslips and ∼600 cells for cytospins.

In a next step, the LA-ICP-TOFMS analysis of adherent macrophage cell models at the single-cell level was validated. To generate cytospin samples from adherent cell models, the sample preparation includes trypsinization followed by cytocentrifugation for cell deposition. The use of chambered glass coverslips offers the possibility to analyze adherent cell models directly, omitting detaching and subsequent deposition. As a major drawback, cell growth potentially expands into three dimensions at high densities. Thus, the production of nicely separated cells in monolayers is not straightforward and needs to be carefully optimized for each in vitro model. Moreover, adhesion/migratory behavior of different cell types often results in complex contours of cells on the chambered glass coverslips (Figures 2A and S1). Both aspects challenge accurate automated data evaluation/cell segmentation. Samples of M0 macrophages incubated with cisplatin were prepared following the two complementary sample preparation strategies. LA-ICP-TOFMS analysis revealed excellent agreement of total platinum amounts in single cells (Figure 2; 25–75 percentile for chamber slides: 0.63–3.07 fg cell^–1^, for cytospins: 0.89–3.13 fg cell^–1^). In addition, a Welch Two Sample *t*-test was performed, which resulted in a *p*-value of 0.365, therefore, no significant difference could be seen between those two sample groups. As automated cell segmentation, a prerequisite for large-scale studies, is facilitated by samples prepared by cytospinning, the method was preferred over chambered glass coverslips.

### Exclusion of Dead Cells after Rh-Labeling

Reliable identification of dead cells is of major importance for data interpretation in mass cytometric analysis.^58^ The gold standard for live/dead cell staining in mass cytometry is the incubation of cells with a high concentration of cisplatin for a short time. The intact membrane represents a physical barrier for the short exposure to cisplatin, whereas the diffuse and leaky membrane of dead cells allows the incorporation of the compound even at this short time scale.^59^ This established marker could not be used in this study as the uptake of cisplatin in cells of the innate immune system was investigated. Alternatively, a recently introduced Rh intercalator^60^ was evaluated as a live/dead cell marker in a proof-of-principle experiment using THP-1 cells. For this purpose, THP-1 monocytes were manipulated by heat-killing prior to labeling with the Rh intercalator. Samples of non-heat-treated THP-1 cells, a 50:50% mixture of viable and heat-killed cells, and 100% heat-killed cells were analyzed by LA-ICP-TOFMS (Figure S2). The ^31^P^+^ signal (red) was used to visualize individual cells, whereas an overlay with the ^103^Rh^+^ signal (green) identified dead cells. Without heat treatment, only a few cells could be identified as dead cells (Figure S2A), whereas in sample B, roughly half of the cells were labeled with Rh (Figure S2B) and in sample C almost all cells contained Rh, indicating the presence of dead cells (Figure S2C). Upon comparison of bright-field images and Rh intensity maps, it was observed that dead cells could be already identified based on bright-field images only (some exemplary dead cells are marked with a red circle), due to the fact that cells displaying a high Rh signal (marked in green) correlated with either ‘black spots’ or cell debris.

The validation experiment was expanded to the macrophage subtypes M0, M1, and M2. All samples including the monocytic cell line THP-1 and the M0, M1, and M2 macrophages were incubated with the Rh intercalator prior to fixation and analyzed by LA-ICP-TOFMS measurements (Figure S3), confirming the observation that dead cells could be identified based on microscopy only (exemplary dead cells are marked with a red circle). Summarizing, for LA-ICPMS analysis, the number of sample preparation steps can be minimized by omitting labeling of dead cells and using cell size markers due to the orthogonal information provided by bright-field images and the measurement of endogenous phosphorus. However, when comparing THP-1 cells with M0, M1, and M2 macrophages, a clear overlay of a high platinum content with the cell death marker could be observed only in the case of the THP-1 monocytes but not for the differentiated macrophages. This difference can be explained by the nonadherent and highly proliferative character of the monocytes and the adherent and nonproliferative character of the macrophages, as well as the sample preparation steps included. At the beginning of an experiment, THP-1 cells are centrifuged, counted, and set up in the respective wells for cisplatin exposure. This implicates that dead cells, which are always naturally present in a proliferative cell culture, remain in the experimental group and are exposed to cisplatin. In contrast, in the case of adherent macrophage cultures, the medium together with dead and floating cells is removed before cisplatin exposure. Therefore, in the case of THP-1 cells, dead cells will be present at the beginning of the 6 h cisplatin exposure, while they were removed in the macrophage samples. In the case of macrophages, cell death might be induced later (after cisplatin exposure) by the more stressful handling procedure including, e.g., cell trypsinization and washing. The cell death stain is always added at the very last step in the cell solution before cytospin preparation. Accordingly, cells dying during the cell preparation for cytospin (mainly in the adherent M0, M1, and M2 groups) are death stain-positive but comparably low in platinum, as they were still alive during cisplatin exposure, while in THP-1 cells, the dead cells unspecifically bound high platinum levels. This hypothesis is also supported by the morphology of the dead THP-1 cells on cytospin, which seems distinctly different (small and highly condensed) from that observed in the M0, M1, and M2 macrophages (flatter and more extended).

### Comparison of THP-1 Monocytes and M0, M1, and M2 Macrophages

Finally, the established and validated single-cell LA-ICP-TOFMS workflow was applied to study the quantitative cisplatin uptake depending on the differentiation and polarization state of monocytes and macrophages. Monocytes can differentiate into macrophages upon specific signals, and depending on the microenvironment and signaling pattern, these M0 macrophages can further be polarized into a variety of subtypes with the most prominent ones being designated as M1 and M2 macrophages. These subtypes differ in physiology, activity, and function. The anti-inflammatory M2 state is associated with enhanced tumor growth, angiogenesis, metastasis, and resistance of tumor cells to chemotherapy, whereas the proinflammatory M1 phenotype rather induces tumor regression and support anticancer immunity. In the tumor microenvironment, TAMs have been described to include both phenotypes, but immune suppression by M2-like macrophages often dominates. Consequently, macrophages and their role within the tumor (immune) microenvironment are a current topic in cancer therapy and M2 macrophages are considered as a therapeutic target.^61−63^

**Figure 3:**
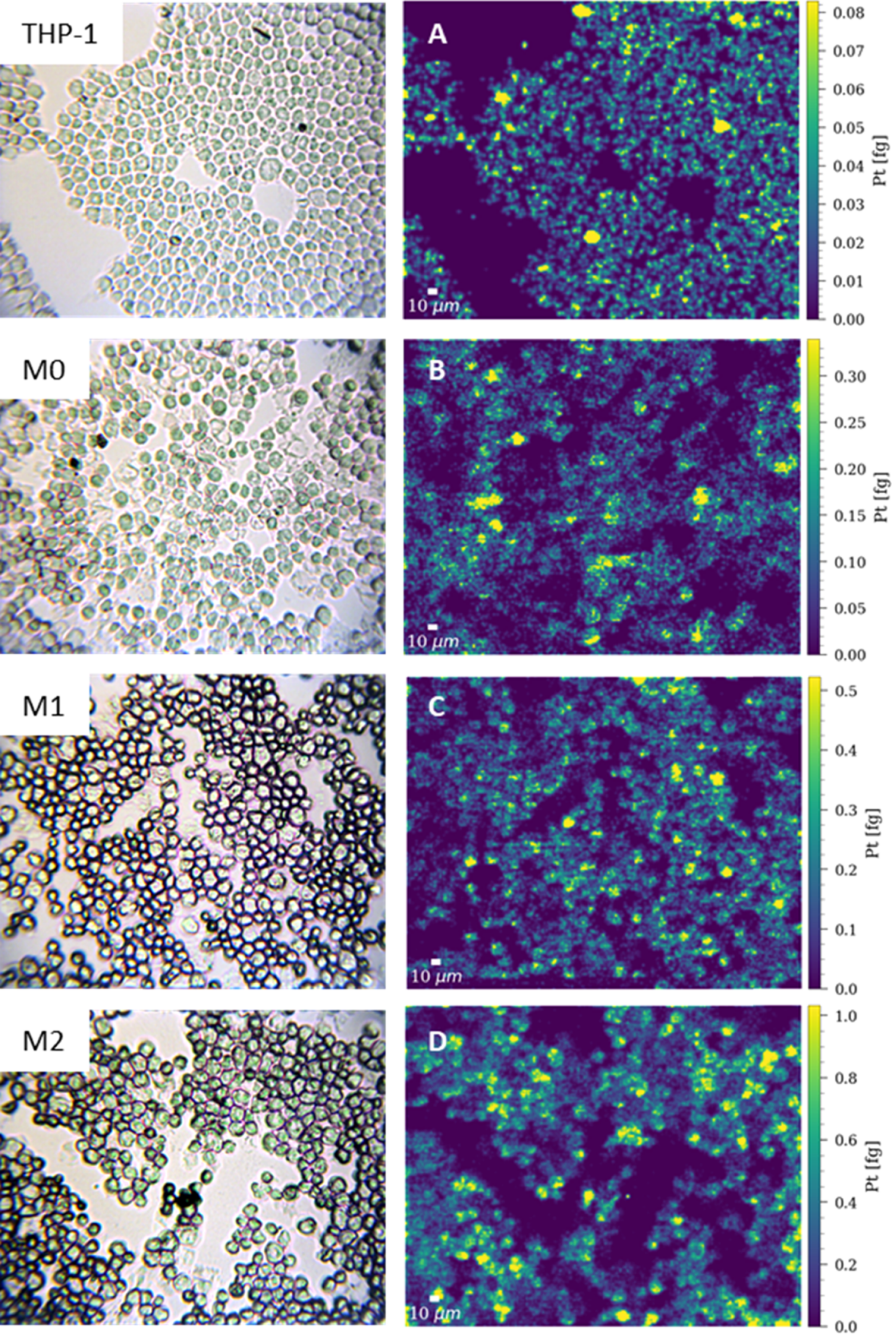
Bright-field images of THP-1, M0, M1, and M2 cells prepared by cytospins prior to ablation (left row). Maps of the total amount of Pt (fg) in (A) THP-1, (B) M0, (C) M1, and (D) M2 cells after treatment with 10 μM cisplatin for 6 h obtained by LA-ICP-TOFMS imaging (right row). The following laser ablation parameters were used: square laser spot size of 5 μm, fixed dosage mode of 2, repetition rate of 200 Hz, and the parallel lines overlapped one another by 2.5 μm. The images were scaled for the best contrast and not for a uniform scale of the Pt concentration.

**Figure 4:**
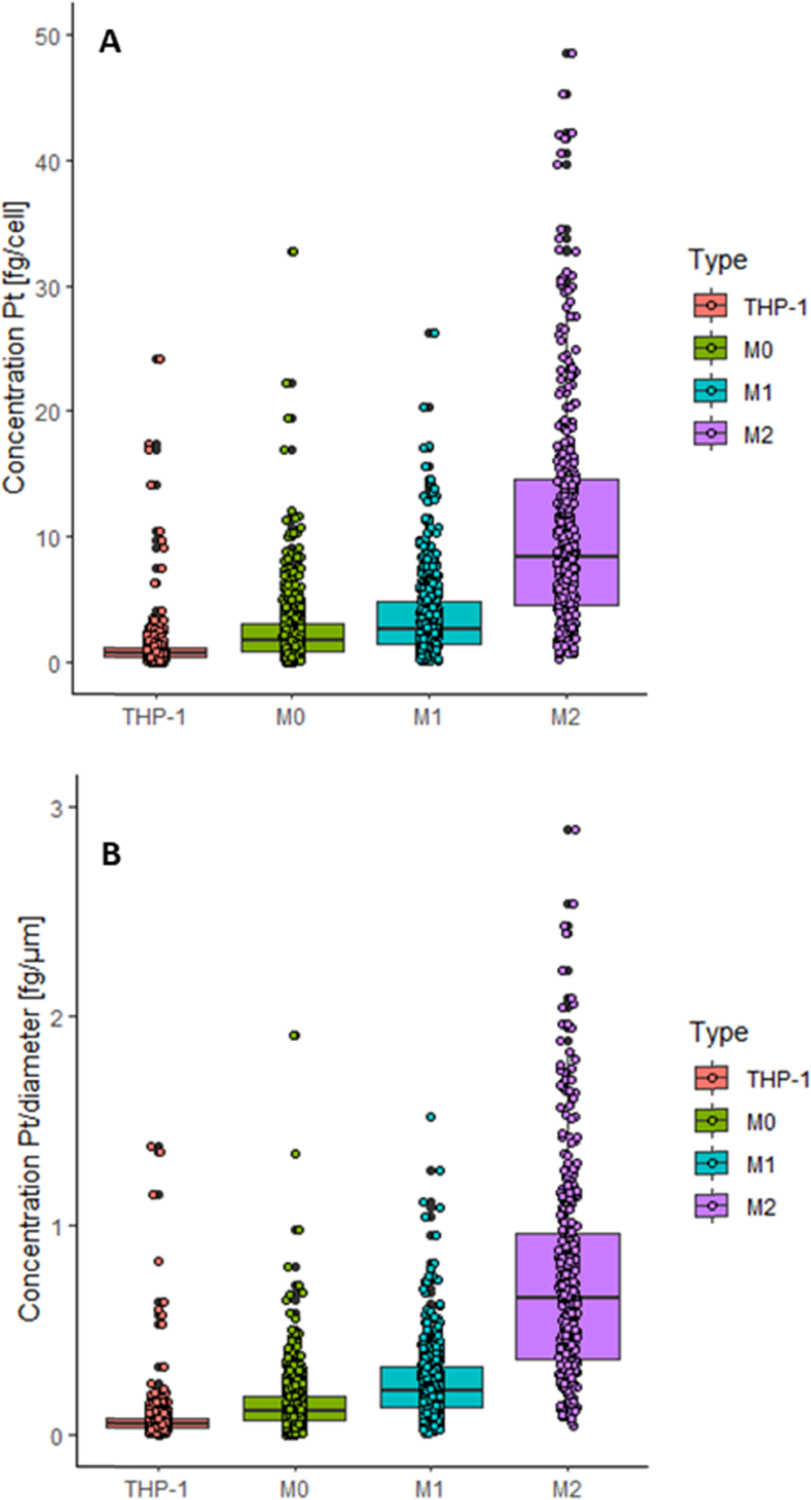
Box plots showing (A) the concentration of Pt cell^–1^ and (B) the concentration of Pt normalized by the cell size of single THP-1, M0, M1, and M2 cells treated with 10 μM cisplatin for 6 h and measured by LA-ICP-TOFMS. The following laser ablation parameters were used: square laser spot size of 5 μm, fixed dosage mode of 2, repetition rate of 200 Hz, and the parallel lines overlapped one another by 2.5 μm. The results are based on ∼900 cells for THP-1 monocytes, ∼600 cells for M0 and M1, and ∼400 cells for M2 macrophages.

Figure 3 illustrates an increasing platinum incorporation starting with the lowest concentration in THP-1 cells, followed by M0 and M1 cells and the highest Pt levels in M2 cells upon cisplatin treatment. These results are further confirmed by box plots (Figure 4A; based on several hundred cells for each cell line) and are summarized in Table S2, with 0.44–1.18 fg cell^–1^ for THP-1 cells, 0.89–3.13 fg cell^–1^ for M0, 1.45–4.79 fg cell^–1^ for M1, and 4.55–14.60 fg cell^–1^ for M2 (25–75 percentile). The segmentation of single cells was based on bright-field images, and the segments were transferred to the laser ablation images after aligning the bright-field images with the signal intensity maps of ^31^P^+^ (Figure S4). To exclude the effect of the cell size on the Pt levels, the Pt concentration of the cells was normalized by the cell size. The size of the cells was assessed by the number of pixels of a single cell and then converted into the equivalent diameter in micrometers. All cell types showed a similar size in the range of ∼15 μm diameter (Figure S5), and therefore, it could be concluded that the observed differences in Pt accumulation between the investigated cell lines were not cell size dependent (Figure 4B).

The most significant increase of platinum incorporation at the single-cell level was seen in M2 macrophages in comparison to the other investigated cell types, while THP-1 cells and M0 and M1 macrophages showed a lower difference relative to each other. The increased uptake detected in the M2 population might explain the cisplatin hypersensitivity of this cell type described in the literature.^64−66^ However, within this study, we exclusively aimed at analyzing drug uptake and therefore chose a short exposure time (6 h) to avoid cisplatin-related cell death. Interestingly, not only the average Pt concentration in the four different cell types increased from THP-1 monocytic cells to M0, M1, and M2, but M2 macrophages also showed the highest degree of variability in Pt concentrations.

Even though no drug-related cell death could be investigated during this short exposure time, where cell death is reflecting a spontaneous event and not a drug effect, a highly increased degree of DNA damage could already be observed in M2 macrophages in comparison to M1 macrophages (see Figure S6). These results were in accordance with the higher Pt incorporation of M2 macrophages leading to a more severe DNA damage. However, also based on literature data,^64^ it is very likely that the cell death rate will be increased, especially in M2 macrophages based on higher drug uptake, after a longer incubation time. Moreover, THP-1 monocytic cells are highly proliferative, whereas upon differentiation into macrophages, the cells become adherent and cease to proliferate. As DNA replication in the presence of cisplatin-DNA-cross-links is boosting double-strand breaks and apoptosis induction, the comparison of proliferative THP-1 with nonproliferative M0, M1, and M2 macrophages specifically for drug-induced cell death induction is generally challenging.

## Conclusions

The established single-cell LA-ICP-TOFMS workflow is characterized by the quantitative analysis of hundreds of cells within minutes, allowing statistical evaluation of a heterogeneous cell population. Cross-validation of the method was achieved by the quantitative analysis of cells in suspension using ICP-TOFMS. Cytospins proved to be a straightforward sample preparation strategy generating nicely separated and evenly distributed cells on a confined area and therefore permitting automated data evaluation. Automation of single-cell segmentation was further facilitated by the high-resolution of the approach and the endogenous elemental pattern of phosphorus and sodium, as measured by LA-ICP-TOFMS. Importantly, single-cell analysis by LA-ICP-TOFMS allows the direct assessment of the cell size and morphological parameters, based on microscopic images without the need for additional labeling approaches. Prior to data evaluation, apoptotic cells, cell debris, or cell clusters can therefore be easily excluded. Proof-of-principle experiments of the developed method on the cisplatin uptake in monocytes and macrophages revealed high Pt incorporation in M2 macrophages compared to the THP-1, M0, and M1 cell line. Additionally, a highly increased DNA damage could be observed in M2 macrophages in comparison to M1 macrophages. This observation supported previous reports on M2 polarized macrophages, which were found to be hypersensitive against metal-based anticancer drugs. The developed single-cell analysis LA-ICP-TOFMS approach can be expanded to study the quantitative uptake of other metallodrugs or nanoparticles at the single-cell level.

## Supporting Information Available

Description of cell culture experiments, instrumental parameters for ICP-TOFMS measurements, bright-field images of M1 and M2 macrophages based on chambered glass coverslips, bright-field images and ^31^P^+^/^103^Rh^+^ overlay of heat-killed and live/death-stained THP-1 cells, ^195^Pt^+^ and ^103^Rh^+^ overlay of cisplatin-treated and live/death-stained monocytes/macrophages, bright-field images and signal intensity maps of ^31^P^+^ in THP-1, M0, M1, and M2, Pt concentrations of THP-1, M0, M1, and M2 cells treated with cisplatin, box plots showing the equivalent diameter of THP-1 and M0, M1, and M2 macrophages, and ^195^Pt^+^ and ^165^Ho^+^ overlay of cisplatin-treated and pH2Ax-stained (DNA damage) M1 and M2 (PDF)
