# Supplementary material for: Cisplatin Uptake in Macrophage Subtypes at the Single-Cell Level by LA-ICP-TOFMS Imaging
Source: Anal Chem. 2021 Nov 30;93(49):16456–65. doi: 10.1021/acs.analchem.1c03442 (PMC8674877; doi:10.1021/acs.analchem.1c03442)
Supplement: Supplementary file 1 — ac1c03442_si_001.pdf [file ac1c03442_si_001.pdf]

Supporting Information

Cisplatin uptake in macrophage subtypes at the single-cell level by LA-ICP-TOFMS imaging

Anna Schoeberl[1], Michael Gutmann[2], Sarah Theiner[1*], Martin Schaier[1], Andreas Schweikert[1,3], Walter Berger[2], Gunda Koellensperger[1*]

[1] Institute of Analytical Chemistry, Faculty of Chemistry, University of Vienna, Waehringer Strasse 38, 1090 Vienna, Austria

[2] Institute of Cancer Research and Comprehensive Cancer Center, Medical University of Vienna, Borschkegasse 8A, 1090 Vienna, Austria

[3] Institute of Inorganic Chemistry, Faculty of Chemistry, University of Vienna, Waehringer Strasse 42, 1090 Vienna, Austria

* Email: sarah.theiner@univie.ac.at, Tel: +43 1 4277 52383

* Email: gunda.koellensperger@univie.ac.at, Tel: +43 1 4277 52303

Table of Content

S2-S3. Experimental S1. Description of cell culture experiments.

S4. Table S1. Instrumental parameters for ICP-TOFMS measurements.

S5. Figure S1. Bright field images of M1 and M2 macrophages based on chambered glass cover slips.

S6. Figure S2. Bright field images and $^{31}P^{+}$/$^{103}Rh^{+}$ overlay of heat-killed and live/death-stained THP-1 cells.

S7. Figure S3. $^{195}Pt^{+}$ and $^{103}Rh^{+}$ overlay of cisplatin-treated and live/death-stained monocytes/macrophages.

S8. Figure S4. Bright field images and signal intensity maps of $^{31}P^{+}$ in THP-1, M0, M1 and M2.

S9. Table S2. Pt concentrations of THP-1, M0, M1 and M2 cells treated with cisplatin.

S10. Figure S5. Box plots showing the equivalent diameter of THP-1 and M0, M1 and M2 macrophages.

S11. Figure S6. $^{195}Pt^{+}$ and $^{165}Ho^{+}$ overlay of cisplatin-treated and pH2AX-stained (DNA damage) M1, M2.

Experimental S1. Cell culture

The human monocytic cell line THP-1, obtained from the American Type Culture Collection (ATCC) (Rockville, MD, USA) was seeded in 6-well plates (for cell suspensions and cytospins) or in chambered glass coverslips (Ibidi, Gräfelfing, Germany) and maintained in Roswell Park Memorial Institute (RPMI) 1640 (Sigma-Aldrich) culture medium supplemented with 10% fetal calf serum (PAA, Linz, Austria) at 37 °C and 5% CO_2 in a humidified tissue culture incubator. THP-1 monocytes were differentiated into macrophages (M0) using 24 h incubation with *phorbol-12-myristat-13-acetat* (PMA) (75 nM). To achieve classically activated macrophages (M1), cells were incubated in medium supplemented with interferon-γ (INF-γ, 20 ng ml^{-1}) and lipopolysaccharide (LPS, 1 ng ml^{-1}) for 24 h. M0 macrophages were polarized into M2 macrophages by incubation with interleukin 4 (IL-4, 25 ng mL^{-1}) and IL-13 (25 ng mL^{-1}) for 48 h. All cells (THP-1, M0, M1 and M2) were recovered for 24 h and then treated with 10 µM cisplatin for 6 h. To prepare cytospins, cells were detached using TrypLE™ Express, washed twice with TBS, centrifuged with a Cytospin 4 centrifuge (Thermo Scientific) at 350 rpm for 5 min and transferred onto glass slides (Superfrost) using a cytocentrifuge. The glass slides were dried at RT, fixed with 4% PFA and washed with water. The chambered glass coverslips (M0) were washed twice with TBS, fixed with 4% PFA, washed twice with water and dried at RT. For live/dead staining, the Cell-ID Intercalator-103Rh was added after cisplatin treatment at a concentration of 1 µM for 30 min[48] and cytospins were prepared as described above. For validation of live/dead staining, an aliquot of THP-1 cells was heat killed at 55 °C for 25 min. Subsequently, three different THP-1 samples containing 0%, 50% or 100% dead cells were prepared. To study the caused DNA damage on the macrophages, cytospins of fixed M1 and M2 were labelled with the DNA damage marker anti-pH2AX (containing ^{165}Ho). Therefore, the slides were first incubated for 30 min in an antigen retrieval solution (96 °C), washed twice with water, permeabilized with 0.2% Triton X-100 in TBS and washed twice with TBS. The permeabilization was followed by a blocking step with 3% BSA in TBS for 45 min at RT. The cytospins were then incubated over night with the anti-pH2AX antibody (in 0.5% BSA) at 4 °C, washed twice with TBS and stained with the Cell-ID Intercalator-191/193Ir (diluted to

a final concentration of ~ 0.3 μM) for 30 min at RT. As a final step, the slides were washed in water and air-dried at RT.

SC-ICP-TOFMS in suspension was used for validation of the LA-ICP-TOFMS experiments. For this purpose, an aliquot of THP-1 cells was fixed with 4% PFA in solution and stained with a Cell-ID Intercalator-191/193Ir diluted to a final concentration of 0.125 μM in Maxpar® Fix and Perm Buffer (Fluidgm, San Francisco, CA, USA) overnight at 4 °C. The suspension cells were washed three times with ultrapure water and diluted to a final concentration of approximately 10^6 cells mL^{-1} immediately before the measurement.

Table S1. Instrumental parameters for ICP-TOFMS measurements

	LA-ICP-TOFMS	SC-ICP-TOFMS
RF Power [W]	1440	1550
Sampling depth [mm]	3.5	5.3
Cone materials	Ni	Ni
Plasma gas flow [L min^{-1}]	14	14
Auxiliary gas flow [L min^{-1}]	0.80	0.80
Nebulizer gas flow [L min^{-1}]	~0.90	0.40
Additional gas flow [%]	-	45
Measurement mode	standard mode	standard mode
Sample introduction system	Aerosol rapid introduction system (ARIS)	Single Cell System from Glass Expansions
m/z range	14-256	14-256
Dwell time [ms]	5	3
Sample flow rate [µL min^{-1}]	-	10

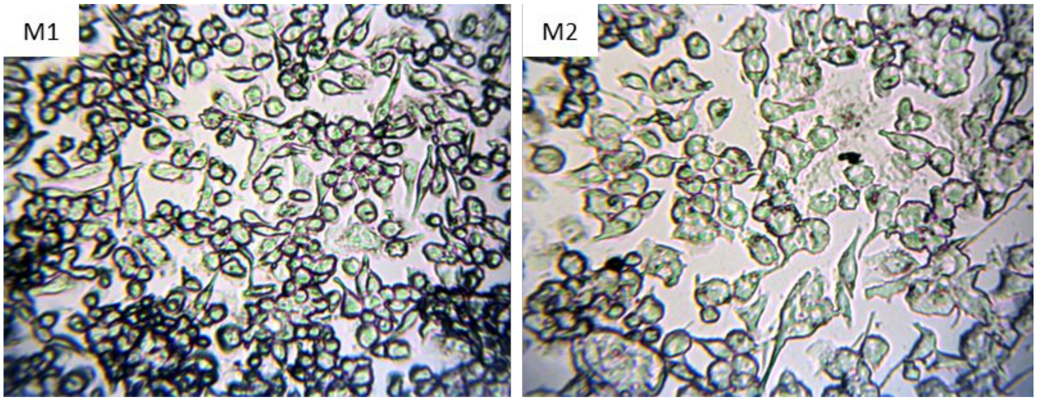

Figure S1. Bright field images of M1 and M2 macrophages based on chambered glass cover slips.

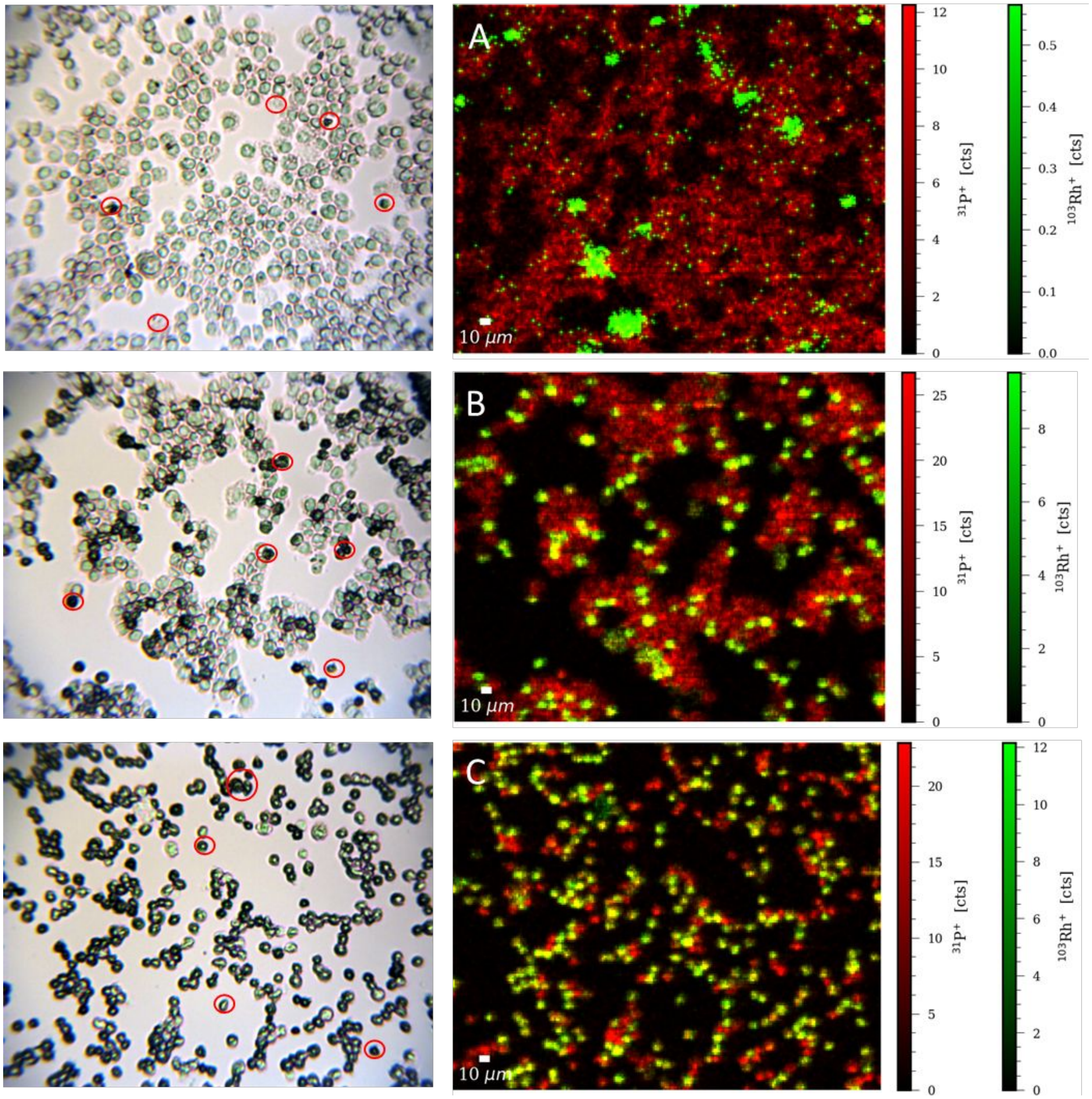

Figure S2. Bright field images of THP-1 cells (left row) prior to ablation. The cells were treated with cisplatin (10 µM, 6 h) and labeled with a ^{103}Rh-intercalator for live/dead cell staining. A varying percentage of cells was heat-killed prior to live/dead cell staining by heating the cells to 55 °C for 25 min: (A) 0% heat-killed cells, (B) 50% heat-killed cells and (C) 100% heat-killed cells. Overlay of the $^{31}P^+$ (red) and $^{103}Rh^+$ (green) signal intensity maps (right row) obtained by LA-ICP-TOFMS imaging. A high Rh signal represents a dead cell. The following laser ablation parameters were used: square laser spot size of 5 µm, fixed dosage mode of 2, repetition rate of 200 Hz, and the parallel lines overlapped one another by 2.5 µm. A few exemplarily dead cells are marked with a red circle.

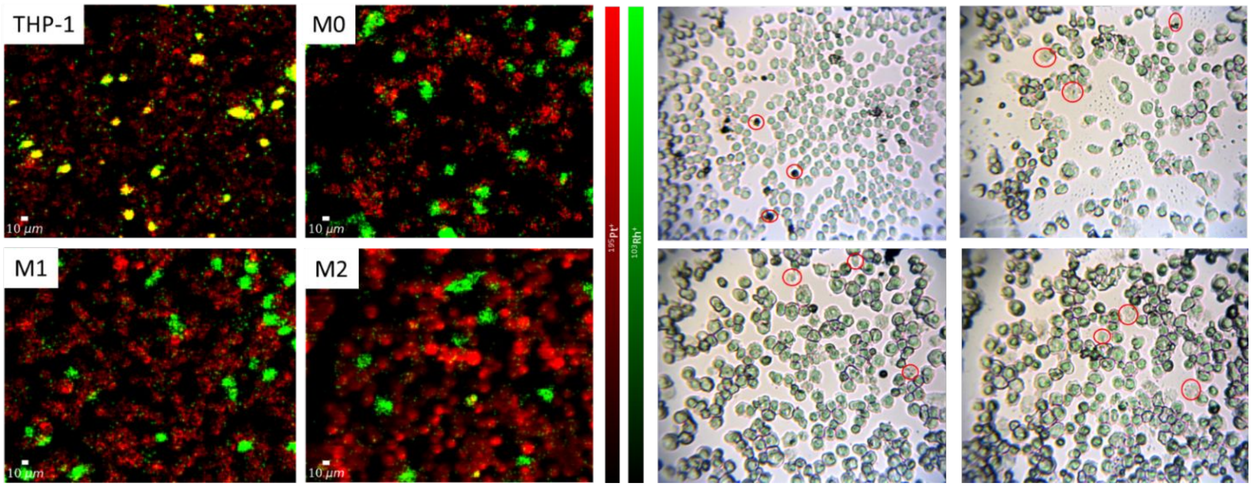

Figure S3. (Left side) Overlay of the $^{195}Pt^{+}$ (red) and $^{103}Rh^{+}$ (green) signal intensity maps obtained by LA-ICP-TOFMS imaging of THP-1, M0, M1 and M2 cells treated with cisplatin (10 µM, 6 h) and labelled with a ^{103}Rh-intercalator for live/death staining. A high $^{103}Rh^{+}$ signal (green) represents a dead cell. The following laser ablation parameters were used: square laser spot size of 5 µm, fixed dosage mode of 2, repetition rate of 200 Hz, and the parallel lines overlapped one another by 2.5 µm. Bright field images of THP-1, M0, M1 and M2 cells prepared by cytospins prior to ablation (right side). A few exemplarily dead cells are marked with a red circle.

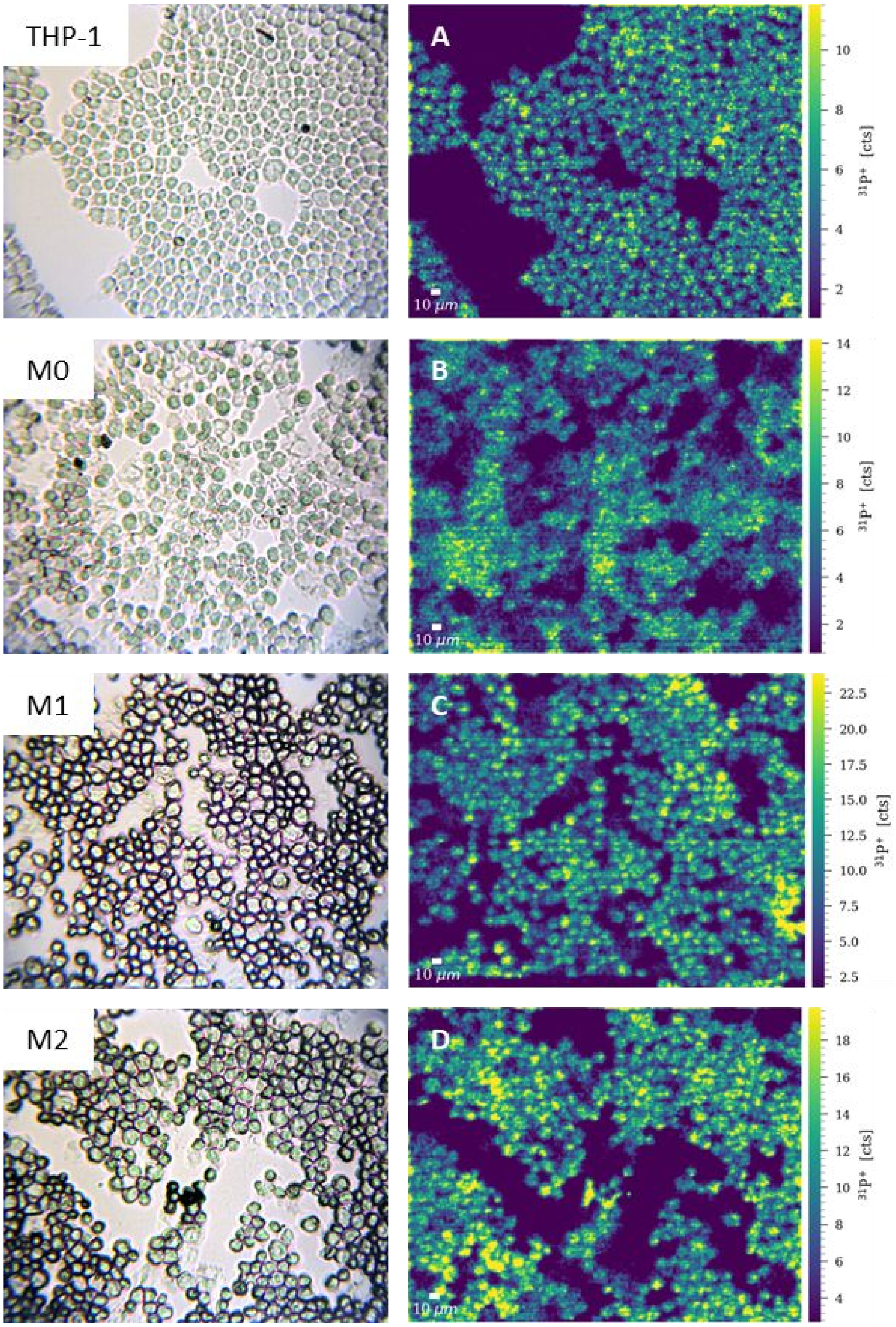

Figure S4. Bright field images of THP-1, M0, M1 and M2 cells prepared by cytospins prior to ablation (left row). Signal intensity maps of $^{31}P^{+}$ in THP-1 (**A**), M0 (**B**), M1 (**C**) and M2 cells (**D**) after treatment with 10 µM cisplatin for 6 h obtained by LA-ICP-TOFMS imaging (right row). The following laser ablation parameters were used: square laser spot size of 5 µm, fixed dosage mode of 2, repetition rate of 200 Hz, and the parallel lines overlapped one another by 2.5 µm.

Table S2. Platinum concentrations of THP-1, M0, M1 and M2 cells treated with 10 μM cisplatin for 6 h measured by LA-ICP-TOFMS, including average, median, 25th percentile and 75th percentile. The following laser ablation parameters were used: square laser spot size of 5 μm, fixed dosage mode of 2, repetition rate of 200 Hz, and the parallel lines overlapped one another by 2.5 μm. The results are based on ~ 900 cells for THP-1 monocytes, ~ 600 cells for M0 and M1 and ~ 400 cells for M2 macrophages.

	Concentration Pt [fg cell^{-1}]			
Parameter	THP-1	M0	M1	M2
Average	0.98	2.47	3.53	10.78
25th percentile	0.44	0.89	1.45	4.55
Median	0.77	1.74	2.68	8.38
75th percentile	1.18	3.13	4.79	14.60

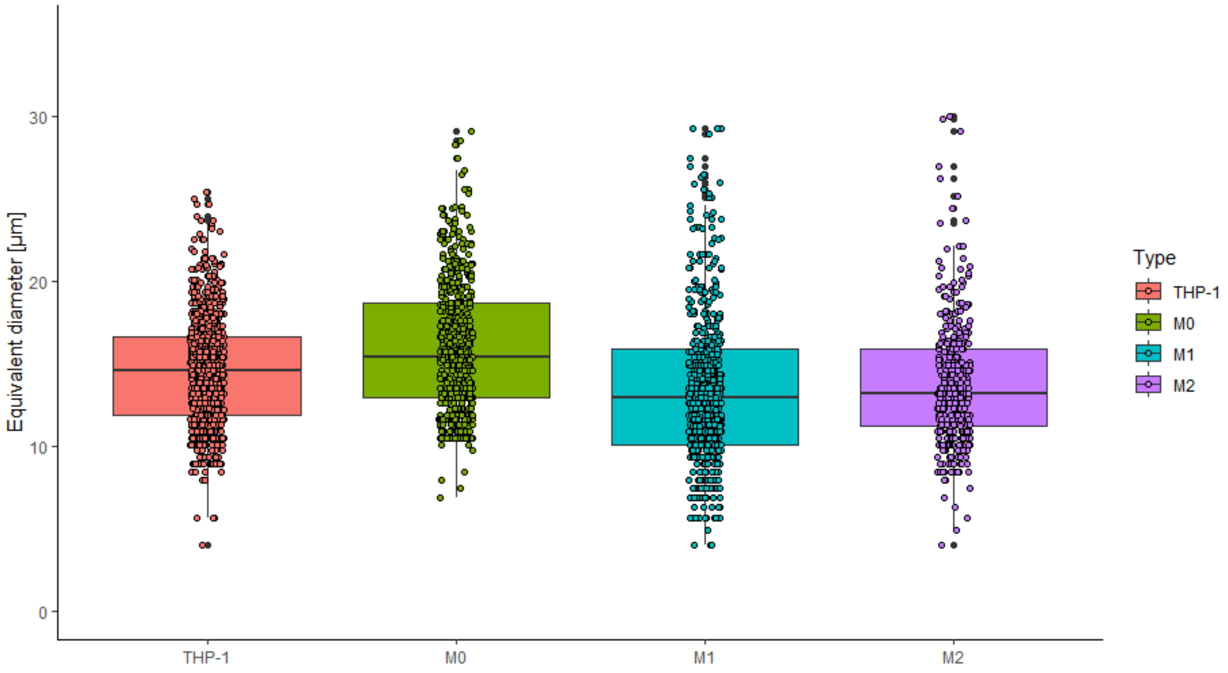

Figure S5. Box plots showing the equivalent diameter of THP-1 monocytes and M0, M1 and M2 macrophages as accessed by LA-ICP-TOFMS analysis. The following laser ablation parameters were used: square laser spot size of 5 µm, fixed dosage mode of 2, repetition rate of 200 Hz, and the parallel lines overlapped one another by 2.5 µm. The results are based on ~ 900 cells for THP-1 monocytes, ~ 600 cells for M0 and M1 and ~ 400 cells for M2 macrophages. The equivalent diameter was accessed by the number of pixels summed up for a single cell.

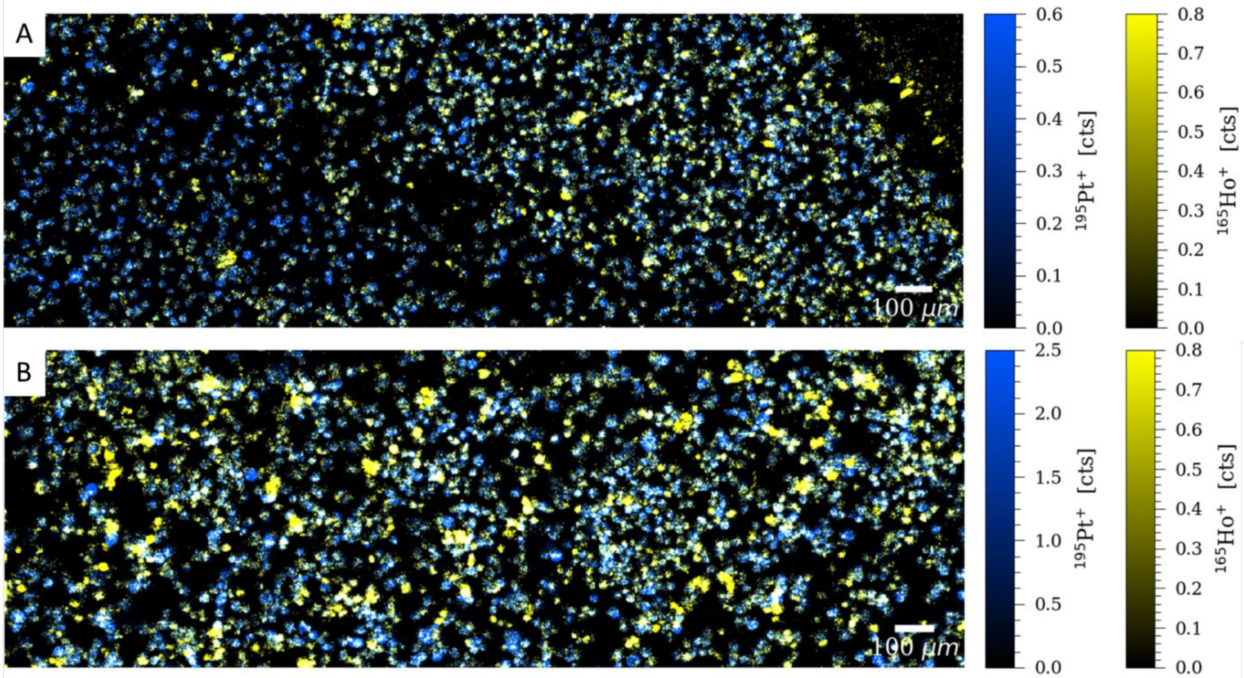

Figure S6. Overlay of the $^{195}Pt^+$ (blue) and $^{165}Ho^+$ (yellow) signal intensity maps by LA-ICP-TOFMS imaging of M1 (**A**) and M2 (**B**) macrophages treated with cisplatin (10 µM, 6 h) and labelled with an antibody against pH2Ax (containing ^{165}Ho) to visualize DNA damage. The following laser ablation parameters were used: circular laser spot size of 4 µm, fixed dosage mode of 2, repetition rate of 200 Hz, and the parallel lines overlapped one another by 2 µm.
